# Tannat Grape Skin: A Feasible Ingredient for the Formulation of Snacks with Potential for Reducing the Risk of Diabetes

**DOI:** 10.3390/nu14030419

**Published:** 2022-01-18

**Authors:** Adriana Maite Fernández-Fernández, Eduardo Dellacassa, Tiziana Nardin, Roberto Larcher, Cecilia Ibañez, Dahiana Terán, Adriana Gámbaro, Alejandra Medrano-Fernandez, María Dolores del Castillo

**Affiliations:** 1 Departamento de Ciencia y Tecnología de Alimentos, Facultad de Química, Universidad de la República, General Flores 2124, Montevideo 11800, Uruguay; afernandez@fq.edu.uy (A.M.F.-F.); mariacecilia.ibsi@gmail.com (C.I.); dahianateran1994@gmail.com (D.T.); agambaro@fq.edu.uy (A.G.); amedrano@fq.edu.uy (A.M.-F.); 2 Instituto de Investigación en Ciencias de la Alimentación (CIAL) (CSIC-UAM), Campus de la Universidad Autónoma de Madrid, C/Nicolás Cabrera, 9, 28049 Madrid, Spain; 3 Graduate Program in Chemistry, Facultad de Química, Universidad de la República, General Flores 2124, Montevideo 11800, Uruguay; 4 Departamento de Química Orgánica, Facultad de Química, Universidad de la República, General Flores 2124, Montevideo 11800, Uruguay; edellac@fq.edu.uy; 5 Dipartimento Alimenti e Trasformazione, Centro Trasferimento Tecnologico, Fondazione Edmund Mach di San Michele all’Adige, Via E. Mach, 1 38010 San Michele all’Adige, Italy; tiziana.nardin@fmach.it (T.N.); roberto.larcher@fmach.it (R.L.)

**Keywords:** α-amylase, anti-inflammatory, antioxidant, bioaccessibility, diabetes, functional foods, α-glucosidase, sensory analysis, sustainable ingredient, Tannat grape skin

## Abstract

In the present work the feasibility of Tannat grape skin (TGS) as a functional ingredient in the formulation of two snacks (yogurt and biscuits) was studied. The research provided novel information on the effects of the food matrix and digestion process, under simulated human oral gastrointestinal conditions, in the bioaccessibility of TGS bioactive compounds composing of the snacks with health promoting properties (antioxidant, anti-inflammatory, and antidiabetic). TGS polyphenolic profile was analyzed by ultra-high performance liquid chromatography tandem mass spectrometry (UHPLC-MS/MS) finding mainly flavonoids, phenolic acids, and anthocyanins, which may exert antioxidant, anti-inflammatory, and carbohydrase inhibition capacities. TGS digest showed antioxidant and antidiabetic potential compared to the undigested sample (*p* < 0.05). Yogurt and biscuits with TGS were developed with the nutrition claims “no-added sugars” and “source of fiber” and were digested in vitro to evaluate the bioaccessibility of compounds with health promoting properties after food processing and digestion. After in vitro simulation of digestion, bioactive properties were enhanced for control and TGS snacks which may be attributed to the formation/release of compounds with health-promoting properties. Biscuits showed significant increase in ABTS antioxidant capacity and yogurt showed increased α-glucosidase inhibition capacity by the addition of TGS (*p* < 0.05). Polyphenols from TGS and bioactive peptides from snacks which may be released during digestion might be responsible for the observed bioactivities. Consumer’s acceptance of TGS yogurt and biscuits showed scores of 6.3 and 5.1 (scale 1–9), respectively, showing TGS yogurt had higher overall acceptance. Sensory profile assessed by check-all-that-apply + just-about-right (CATA+JAR) showed most of the attributes were evaluated as “just about right”, supporting good food quality. The developed yogurt presented adequate shelf-life parameters for 28 days. TGS yogurt with higher acceptability showed reduced ROS formation (*p* < 0.05) induced by tert-butyl hydroperoxide (1 mM) in CCD-18Co colon cells and RAW264.7 macrophages when pre-treated with concentrations 500–1000 and 100–500 µg/mL of the digests, respectively. Moreover, TGS yogurt digest pre-treatment reduced nitric oxide (NO) production (*p* < 0.05) in lipopolysaccharide (LPS)-induced RAW264.7 macrophages, showing anti-inflammatory potential. Bioactive peptides generated during lactic fermentation and digestion process may be contributors to intracellular effects. In conclusion, yogurt and biscuits with Tannat grape skin addition were obtained with nutrition claims “no-added sugars” and “source of fiber” with the potential to modulate key biochemical events associated with diabetes pathogenesis.

## 1. Introduction

Dietary fiber and polyphenols have long been known for their health benefits, being used in a wide range of foods [1] such as bakery, dairy, meat, and drink food products, among others. They have also been incorporated into different food products for their technological properties (texture, viscosity, among others) and shelf-life improvement [2,3,4].

Yogurt is the most popular dairy product with also high nutritional value [4]. However, yogurt lacks the dietary fiber and polyphenols being commonly mixed with fruits [4] or fortified using plants/fruits in order to improve bioactive properties [5,6,7]. In the same way, bakery products such as biscuits are consumed daily in large quantities, comprising a significant role in human nutrition and thus, also represent an ideal food product for the addition of functional ingredients [8] such as winemaking byproducts. In particular, Tannat grapes possess high amounts of anthocyanins [9,10], mostly in the skin [11]. In a previous work, Tannat grape skin was found to present antioxidant, antidiabetic, antiobesity, and anti-inflammatory activities [12]. Thus, Tannat grape skin (TGS) may have the potential to reduce the risk of/treat chronic diseases when added to food products as a sustainable functional ingredient. The increase of the number of people suffering from metabolic disorders such as insulin resistance and overweight, among others, which are promoted by chronic oxidative stress and inflammation [13], could be reduced by polyphenols dietary intake [14,15]. In addition, these compounds may ameliorate the complications associated to these pathologies [16].

However, for polyphenols to exert their biological effect, factors such as the cooking process, which modifies the natural profile of phenolic compounds of the food matrix, should be taken into consideration. The interaction between polyphenols and other components composing the food matrix affect their bioaccessibility and bioavailability [3,17,18]. In contrast, food matrix may also protect some polyphenols, such as anthocyanins, from degradation due to thermal processing [19] and physiological digestion [20,21].

The present work provides novel information on the feasibility of TGS as a health promoting ingredient for the formulation of two sustainable snacks that are highly consumed (yogurt and biscuits). To achieve the goal, sensory quality of the food and the effects of food processing and digestion on the in vitro bioaccessibility of health promoting compounds were assessed.

## 2. Materials and Methods

### 2.1. Materials

All reagents were of reagent grade. For antioxidant assays, reagents were purchased from Sigma-Aldrich (St. Louis, MO, USA): 6-Hydroxy-2,5,7,8-tetramethylchromane-2-carboxylic acid (Trolox), 2,2′-azino-bis (3-ethylbenzothiazoline-6-sulfonic acid) diammonium salt (ABTS), 2,2′-azobis (2-methylpropionamidine) dihydrochloride (AAPH), fluorescein (FL) disodium salt. For carbohydrase enzymatic activity assays, reagents were purchased from Sigma-Aldrich (St. Louis, MO, USA): 4-methylumbelliferyl-α-D-glucopyranoside, α-glucosidase from rat intestine acetone powder, 3,5-dinitrosalicylic acid, α-amylase from human saliva (type IX-A), starch, maltose standard, acarbose. Digestion enzymes were also purchased from Sigma-Aldrich (St. Louis, MO, USA).

The normal human colon fibroblast cell line (CCD-18Co) and RAW264.7 mouse macrophage cells were obtained from American Type Culture Collection (ATCC, Manassas, VA, USA). Rat small intestine epithelial cell line (IEC-6) was kindly provided by the Bioanalytical Techniques Unit (BAT) of the Instituto de Investigación en Ciencias de la Alimentación (CIAL) (Madrid, Spain). Cells were cultivated using Dulbecco’s modified Eagle medium (DMEM), L-Glutamine (1% *v*/*v*) and antibiotics (penicillin and streptomycin 1:1, 1% *v*/*v*) all purchased from Gibco Laboratory (Invitrogen Co., Grand Island, NY, USA), while heat inactivated fetal bovine serum (FBS) (10% *v*/*v*) was purchased from Hyclone (Logan, UT, USA). For cell studies assays, reagents were purchased from Sigma-Aldrich (St. Louis, MO, USA): 3-(4,5-dimethylthiazol-2-yl)-2,5-diphenyltetrazolium bromine (MTT), oxidant-sensitive probe 2′,7′-dichlorofluorescin diacetate (DCFH-DA), sulfanilamide, N-(1-napthyl) ethylenediamine dihydrochloride, phosphoric acid, sodium nitrite and lipopolysaccharide from *E. coli* O55:B5 (LPS).

Food ingredients were purchased from local stores in Montevideo (Uruguay): UHT whole fluid milk, skim milk powder, modified cassava starch, gelatin, CRL inulin (soluble fiber), stevia, wheat flour, sweetener (Sucralose^®^), butter, sunflower oil, egg, and baking powder. YO-MIX 495 LYO ferment (250 DCU) (Prochemie-DANISCO, Montevideo, Uruguay).

### 2.2. Samples

Tannat grape skin powder (TGS) from Tannat grape pomace was provided by Bouza wine cellar (Montevideo, Uruguay) and prepared as previously described [12]. Briefly, seeds and skins of Tannat grape pomace were manually separated, skin was dried at 40 °C for 24 h (up to constant weight) and then powdered using a domestic mill and stored at −20 °C for further analysis.

TGS yogurt and biscuits formulation were designed with the nutrition claims “no-added sugars” and “source of fiber” according to MERCOSUR regulations (>2.5 g of fiber per serving) [22]. TGS was added in a proportion of 0.5% and 20% *w*/*w* in yogurt and biscuits (Table 1), respectively, resulting in a fiber content of 3 g/200 mL of yogurt (inulin and TGS fiber) and 9.46 g/100 g of fresh biscuit dough (TGS fiber). Moreover, the addition of TGS resulted in a non-significant reduction of carbohydrates (10% decrease) in the biscuit formulation as well as of kcal (128.99 kcal from control biscuit to 117.28 kcal of TGS biscuit).

The optimization of the yogurt formulation was tested with the addition of TGS, inulin and stevia making its consumption suitable for diabetic people. Briefly, stirred yogurt formulations were prepared mixing in a MyCook (Taurus) with UHT whole fluid milk, skim milk powder, modified cassava starch, gelatin, CRL inulin (soluble fiber), stevia, and TGS powder (0 and 0.5% *w*/*w*). The procedure was as follows: whole fluid milk was heated at 50 °C in the MyCook for 3 min at speed 3; then skim milk powder, modified cassava starch, gelatin, CRL inulin (soluble fiber), stevia, and TGS powder (0 and 0.5% *w*/*w*) were added to whole fluid milk, and heated at 50 °C for 5 min at speed 3. Once the mixture was homogeneous, it was heated at 90 °C for 5 min and transferred to a previously autoclaved bottle, cooled in a water bath at ambient temperature and placed in an oven at 42 °C. Each bottle containing 400 g of the mix was inoculated with 1 mL of YO-MIX 495 LYO ferment (*Streptococcus thermophilus* and *Lactobacillus delbrueckii* subspecies bulgaricus, 250 DCU) preparation (0.12 g of ferment was weighed, and 10 mL of UHT whole fluid milk was added). The mixtures were incubated in the oven until pH 4.5 was reached (approximately 4 h for 0% yogurt, and 6 h for 0.5 and 1% yogurt). Once the pH was reached, the bottles were placed in a cold water bath, stirred to get a smoothie yogurt and stored at 4 °C. A yogurt with 1% of TGS was also prepared but showed sensory difficulties.

Biscuits containing TGS as food ingredient (20% *w*/*w* on wet dough basis) were prepared according to the formulation shown in Table 1. The mixture was baked for 12 min at 180 °C and the biscuits stored under dry and fresh conditions until analysis.

### 2.3. Methods

#### 2.3.1. Profile of Phenolic Compounds Composing TGS

The TGS phenolic profile was assessed by UHPLC-MS/MS analysis (10 mg/mL solution in H_2_O:MeOH (50:50, *v*/*v*)) as described by Fernández-Fernández et al. [11]. The identification and quantification of phenolic compounds was carried out using a Thermo Ultimate™ 3000 HPLC (Thermo Scientific, Sunnyvale, CA, USA) coupled to a hybrid quadrupole-orbitrap mass spectrometer (Q-ExactiveTM; Thermo Scientific, Bremen, Germany) equipped with heated electrospray ionization (HESI-II). Chromatographic separation was performed as follows: UPLC BEH C18 column 2.1 mm × 100 mm, 1.7 μm particle size (Waters, Milford, MA, USA); mobile phase, water-acetonitrile gradient at a flow rate of 0.3 mL/min. Thermo Scientific™ Dionex™ Chromeleon™ 7.2 Chromatography Data System (CDS) software was used for data acquisition and processing. The peak area was used to define the relative concentration of the phenolic compounds.

#### 2.3.2. Bioaccessibility of Bioactive Compounds

Before the in vitro bioactivity analysis of TGS, TGS yogurt and TGS biscuits, samples were subjected to an in vitro simulation of the human digestion process mimicking the human oral gastrointestinal conditions as described by Hollebeeck et al. [23]. After the simulation, the mix was centrifuged (10,000 rpm, 10 min) obtaining the bioaccessible fraction (supernatant). Bile salts were removed from the bioaccessible fraction by using cholestyramine resin [11]. The desalted samples (digests) were frozen and stored for further analysis.

- Evaluation of the release of bioaccessible antioxidant compounds

The release of antioxidants during digestion was determined by Total Phenolic Content (TPC) using Folin-Ciocalteau method, and the overall antioxidant capacity by ABTS and ORAC-FL assays as described by Fernández-Fernández et al. [12].

TPC was determined as described by Slinkard & Singleton [24] using a gallic acid standard curve (0.05 to 1.0 mg/mL). Samples were prepared in distilled water by triplicate and the results were expressed as mg gallic acid equivalents (GAE)/g digest, and all determinations were performed in triplicate.

ABTS assay was carried out as described by Fernández-Fernández et al. [12], by adding 10 µL of the sample solutions to translucent flat-bottom 96-well plates and 190 µL of ABTS working solution, 10 min incubation and absorbance measure at 750 nm in a Thermo Scientific FC microplate reader. Trolox was used for the standard curve (0.25 to 1.5 mM). Results were expressed as μmol Trolox equivalents (TE)/g digest. Assays were performed in triplicate.

ORAC-FL assay was performed as described by Fernández-Fernández et al. [12], by adding 20 µL of the sample solutions to black flat-bottom 96-well plates, 120 µL of fluorescein working solution and 60 µL of AAPH, followed by 104 min incubation at 37 °C and measuring fluorescence (λ_excitation_ = 485 nm, λ_emission_ = 520 nm) in a Varioskan Lux (Thermo Scientific) microplate reader. Trolox was used for the standard curve (0.1 to 0.8 mM). Results were expressed as μmol TE/g digest. Assays were performed in triplicate.

- Inhibitors of carbohydrases

The bioaccessible inhibitors of carbohydrases, α-glucosidase and α-amylase, were determined as described by Fernández-Fernández et al. [11] to evaluate the antidiabetic activity of TGS, TGS yogurt, and TGS biscuits. Briefly, the inhibition capacity of α-glucosidase was measured by using a fluorescent probe (4-MUF-α-D-glucopyranoside) and measuring fluorescence (λ_excitation_ = 360 nm, λ_emission_ = 460 nm) in a Varioskan Lux (Thermo Scientific) fluorimeter microplate reader. The inhibition capacity of α-amylase was measured by determining the release of reducing sugars from starch hydrolysis through the reaction with dinitrosalicylic acid color reagent and measuring absorbance at 540 nm in a Varioskan Lux (Thermo Scientific) microplate reader. Acarbose (pharmaceutical of reference with probed inhibition capacity) was used for comparison in both assays. The IC50 values were calculated from the dose–response curves for each sample (% Inhibition vs. [Sample or Standard](mg/mL)).

- Assessment of the effects of the bioaccessible compounds in cell models

Firstly, cell viability was performed by MTT assay as described by Fernández-Fernández et al. [12]. Briefly, cells were seeded (20,000 IEC-6, 10,000 CCD-18Co and 80,000 RAW264.7 cells/well) in sterile 96-well plates and incubating for 24 h reaching cell confluence. Different concentrations of TGS yogurt digest (150 µL/well) were placed in each well and incubated for 24 h. Then, MTT reagent (6 mM, 20 µL/well) was added to each well and the plate was incubated for 30 min (RAW264.7 cells) or 3 h (IEC-6 and CCD-18Co cells). Supernatants were removed, 100 µL of DMSO was added to each well, and plates were incubated for 5 min to achieve better homogenization. Cell viability was estimated by measuring absorbance at 570 nm of the wells that were treated with sample solutions normalized to absorbance of non-treated cells (control cells with DMEM) accounting for 100%.

To evaluate the amelioration of intracellular formation of Reactive Oxygen Species (ROS) by TGS yogurt digest three cell lines were employed: IEC-6 (normal rat small intestine epithelial cells), CCD-18Co (normal human colon fibroblast cells) and RAW264.7 (mouse macrophages) cells. The assays were carried out as described by Fernández-Fernández et al. [25]. The tests were carried out by seeding 20,000 IEC-6, 10,000 CCD-18Co and 80,000 RAW264.7 cells/well in sterile 96-well plates and incubating for 24 h reaching cell confluence. Afterwards, 150 µL of different TGS yogurt bioaccessible fraction concentrations were placed in each well and incubated for 24 h. Then, 2 µL of DCFH-DA probe (5 mg/mL in DMSO) were added to each well and incubated for 30 min. Supernatants were removed, cells washed with PBS, and added with the same concentrations of samples (150 µL) for “Pre-treatment” assay or the same concentrations of the samples with the oxidant agent for “Pre-treatment with co-administration” assay. Oxidation was induced using tert-butyl hydroperoxide 1 mM (final concentration in each well) and incubation for 30 min. The intracellular ROS formation was determined by measuring fluorescence using a Varioskan Lux (Thermo Scientific) fluorimeter microplate reader (λ_excitation_ = 485 nm, λ_emission_ = 528 nm). To normalize data by the viable cells number, MTT assay was performed by adding 20 µL/well of MTT (6 mM) reagent and incubating for 30 min (RAW264.7 cells) or 3 h (IEC-6 and CCD-18Co cells).

The bioaccessible anti-inflammatory compound release from TGS yogurt was evaluated as described by Fernández-Fernández et al. [12] measuring the RAW264.7 mouse macrophages production of nitric oxide (NO) under induced conditions (lipopolysaccharide, LPS). Briefly, RAW264.7 mouse macrophages were seeded in sterile 96-well plates (80,000 cells/well) and incubated for 24 h reaching cell confluence. Different concentrations of TGS yogurt digest (150 µL/well) were added to each well, then plates were incubated for 24 h, followed by LPS-induced inflammation with incubation for another 24 h (pre-treatment assay) or followed by addition of the same TGS yogurt digest concentrations and LPS with incubation for another 24 h (pre-treatment and co-administration assay). Negative (DMEM) and positive (DMEM + LPS) controls were also tested. After 24 h incubation, 100 µL of cells supernatants were removed from cells and placed in another 96-well plate to react for 15 min with 100 µL Griess reagent at room temperature. Absorbance was measured at 550 nm to determine macrophages NO production. Standard curve was built using sodium nitrite (0–10 µg/mL).

#### 2.3.3. Yogurt Shelf-Life

Shelf-life for the 2 formulations (control yogurt and TGS yogurts with 0.5% *w*/*w*) was determined by measuring pH and acidity over time, complemented with microbiological analysis. Acidity was expressed as g of lactic acid/100 g of product by titration with 0.1 N NaOH. Before sensory analysis, microbiological studies were performed by determining total coliforms, fungi, and yeasts. Studies were performed for 28 days.

#### 2.3.4. Sensory Analysis

Sensory analyses were carried out by 75 consumers (40% male and 60% female, aged between 18 and 87 years old) recruited at Departamento de Ciencia y Tecnología de Alimentos (Facultad de Química, Universidad de la República, Montevideo, Uruguay). The evaluated samples were the yogurt and the biscuit formulations added with TGS in a proportion of 0.5 and 20% *w*/*w*, respectively. The sensory analysis was assessed through a CATA (check all that apply) + JAR (just about right) and overall acceptance was evaluated on a scale of 1 to 9. The different foods were evaluated according to the frequency of mention of the different analyzed attributes (“taste”, “visual appearance”, “texture”, and “smell”). Yogurt and biscuit samples were served in black plastic cups. Consumers were informed about the addition of TGS to the food product as follows: “yogurt/biscuit with antioxidants and fiber from grape skin, with no-added sugar and with sweetener”.

#### 2.3.5. Statistical Analysis

Results were expressed as means ± standard deviation (SD) (*n* = 3). The statistical differences between mean values were determined by the Tukey test (*p* < 0.05) and *t*-test (*p* < 0.05) using Infostat v. 2015 program (Grupo InfoStat, FCA, Universidad Nacional de Córdoba, Argentina). Sensory statistical analysis was performed using XLStat-Sensory v. 2017 (Addinsoft, New York, NY, USA) program.

## 3. Results and Discussion

### 3.1. TGS Polyphenolic Profile by UHPLC-MS/MS Analysis

TGS was composed of flavonoids (quercetin-3-galacturonide, quercetin, quercetin-3β-D-glucoside, myricetin, and isorhamnetin), phenolic acids (gallic and cis-aconitic acids) and anthocyanins [malvidin-3-pyranoside, malvidin-3-O-(6-p-coumaroyl)glucoside, malvidin-3-(6-acetylglucoside)] (Table 2). Negative ESI results showed quercetin-3-galacturonide and quercetin as the main flavonoids followed by gallic and cis-aconitic phenolic acids. Positive ESI results showed malvidin derivatives as the main anthocyanins, showing malvidin-3-pyranoside having the highest proportion. According to our previous findings [11] of a Tannat grape skin extract, the latter presented higher proportion of quercetin-3-galacturonide and quercetin (0.4236 and 0.3677, respectively) than TGS (0.1190 and 0.1181) and a lower proportion of anthocyanin malvidin-3-pyranoside (0.00886) than TGS (0.02569), showing TGS to be more enriched in anthocyanins than the extract.

### 3.2. Bioaccessibility of Bioactive Compounds from Tannat Grape Skin (TGS)

The bioactive compounds composing Tannat grape skin and the bioaccessible compounds are shown in Table 3. The bioactivity of polyphenols can change during digestion as a result of chemical structure modification, as pH changes, pancreatin, and bile acids effects are detrimental for anthocyanins [27]. Polyphenol chemical structure changes may affect antioxidant capacity [20]. When compared with previous studies, the bioaccessible fraction of *Vitis labrusca* L. grapes showed the highest bioaccessibility for quercetin, and malvidin and cyanidin were more bioaccessible in the gastric phase, although anthocyanins, flavanols, flavonols, hydroxybenzoic and hydroxycinnamic acids were still bioaccessible after in vitro gastrointestinal simulation of digestion [28]. Phenolic compounds from blackberries, including anthocyanins, have also been reported for degrading during digestion [29]. The results in Table 3 are in agreement with the degradation of polyphenols after digestion but with remaining polyphenols [30] with antioxidant capacity.

Results regarding the bioaccessibility of carbohydrase inhibitors showed improved effect on α-glucosidase enzyme after digestion probably because of polyphenol release from Tannat grape skin matrix, which were reported for inhibiting these carbohydrases [31,32,33]. These results are in contrast with our previous findings on digested Tannat grape skin extract [11], meaning dietary fiber may have protected the inhibitors of carbohydrases from digestion degrading conditions. Moreover, some of the anthocyanins composing TGS (Table 2) were reported for being bioaccessible [34,35] and being at least partially responsible of the subsequent in vitro bioactivities.

### 3.3. TGS Yogurt and Biscuit Bioaccessible Compounds

**Figure 1:**
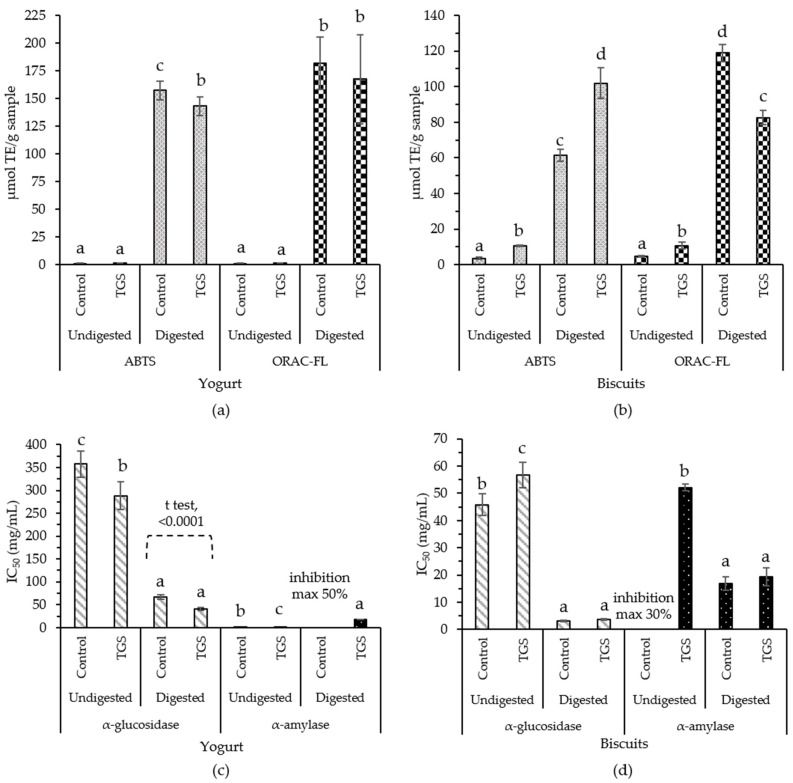
Effects of food processing and digestion on antioxidants and inhibitors of carbohydrases composing snacks formulated with TGS. Results shown in the figure correspond to the data obtained by: ABTS (**a**) and ORAC-FL (**b**) α-glucosidase (**c**) and α-amylase (**d**). Bars and error bars represent mean values and standard deviation. Results on antioxidant capacity are expressed as µmol TE/g sample and the results of enzymatic inhibition capacity are expressed as IC_50_ values (half maximal inhibitory concentration) in mg/mL. Lowest IC_50_ means highest inhibitory capacity. Different letters indicate significant differences (Tukey, *p* < 0.05) between mean values of each set of data. T test was carried out to compare mean values of TGS snacks and their respective control snacks (formulations without TGS).

The presence of bioactive compounds in foods (undigested samples) and the corresponding digests (digested samples) was estimated by ABTS, ORAC-FL (Figure 1a,b), α-glucosidase and α-amylase enzymatic activities (Figure 1c,d). No significant differences (*p* > 0.05) on antioxidant capacity were detected between undigested control yogurt compared with undigested TGS yogurt. Between undigested samples, TGS yogurt presented higher α-glucosidase inhibitory capacity (lower IC_50_) than control yogurt (*p* < 0.05) (Figure 1c) that could be influenced by TGS polyphenols [12].

Other authors have studied yogurts fortified with different natural sources of bioactive compounds. Stirred yogurt fortified with pomegranate peel extracts (5, 10, 15, 20, 25, 30 and 35%), before and after inoculation with the traditional yogurt starter, showed higher antioxidant capacity when extracts were added before inoculation, and fortification was found to increase antioxidant capacity with an increase in concentration up to 25% [36]. Noteworthy, these last studies whose results are not in agreement with the present work, performed higher amounts of fortification in the yogurts with the subsequent higher antioxidant capacity. TGS yogurt containing higher amounts of the ingredient enriched in polyphenols were not possible in our experimental conditions without affecting the sensory quality of the food. Results seem to indicate that the amount of phenols remaining after food processing and digestion is not enough to cause a significant increase (*p* > 0.05) of the overall antioxidant properties of the control yogurt formulation.

Food bioaccessibility studies related to antioxidant capacity of digested TGS yogurt (Figure 1a) demonstrated a decrease with respect to the control yogurt for ABTS value but was maintained for ORAC-FL value (*p* > 0.05), presenting the same tendency as undigested samples. Oliveira & Pintado [37] reported a strawberry and peach enriched yogurt after in vitro gastrointestinal digestion, showed an increment in the radical scavenging capacity when compared to undigested fruit yogurt and a loss in all polyphenol classes at the intestinal phase, still releasing considerable amounts of polyphenols to be absorbed at intestinal level promoting health benefits. The bioaccessibility of TGS polyphenols may be affected due to their degradation or binding to food components such as polysaccharides, lipids, or proteins [3,37]. Moreover, antioxidant capacity of yogurts may be attributed to milk proteins (caseins and whey proteins) which suffer pepsin hydrolysis during digestion, releasing encrypted antioxidant peptides [38]. The α-glucosidase inhibitory capacity of yogurt digest containing TGS (Figure 1c) was significantly higher (lower IC_50_) compared to that found for digested control yogurt (*t* test, *p* < 0.0001), which may be exerted by TGS remaining polyphenols, being in agreement with the results in Table 3. Yogurt digests also showed α-amylase inhibitory capacity (Figure 1c) with no significant differences (*p* > 0.05) between control and TGS yogurts. The displayed inhibitory capacity may have been exerted by the bioactive peptides from milk [39,40].

Regarding biscuits, addition of TGS caused a significant increase (*p* < 0.05) of overall antioxidant capacity of the food (undigested sample) (Figure 1b) which may be associated to TGS polyphenols. Baking process has been reported for having detrimental effects on polyphenol stability in cookies with anthocyanin sources [41], like the one employed in the present work. Still, the addition of these sources to cookies, have shown to increase the nutritional value and antioxidant capacity when compared to the cookie without anthocyanin source addition [42], being in agreement with the current results.

A positive effect on the antioxidant capacity of the digests (Figure 1b) was observed for the TGS biscuits using the ABTS method when compared to control biscuit (*p* < 0.05). Bioaccessible compounds (digested samples) with α-glucosidase inhibitory capacity in TGS biscuits (Figure 1d) showed no significant differences (*p* > 0.05) with control biscuits after in vitro simulation of digestion, which may be a consequence of impairing TGS polyphenols bioactive properties by the interaction with polysaccharides, proteins, and lipids that compose the ingredients of the biscuit [43] or polyphenol degradation during digestion [37]. Moreover, the α-amylase inhibitory capacity assay showed no significant differences between the TGS biscuit and the control (*p* > 0.05), still with remaining bioaccessible α-amylase inhibitors. The observed inhibition capacity may be due to the biscuits’ ingredients, such as wheat proteins [44], released during digestion. In addition, phenolic acids released from wheat during digestion might also be responsible for the exerted bioactivities [44].

Overall, the digested yogurts presented more antioxidant capacity than the digested biscuits, which may be attributed to yogurt bioactive peptide release during digestion [38]. Polyphenols from TGS may interact with macromolecules from the snack ingredients, affecting their biological effects [3,37,43,45].

To the best of our knowledge, this is the first report on bioaccessible antioxidants and inhibitors of carbohydrases from TGS sustainable snacks. Moreover, bioaccessibility studies of TGS yogurt and biscuits ensuring bioactivity after in vitro digestion and the simplicity of TGS powder processing for being used as a functional ingredient, makes it accessible for scaling by industry and a valuable asset for the innovative development of healthy sustainable snacks.

### 3.4. Yogurt Shelf-Life

**Figure 2:**
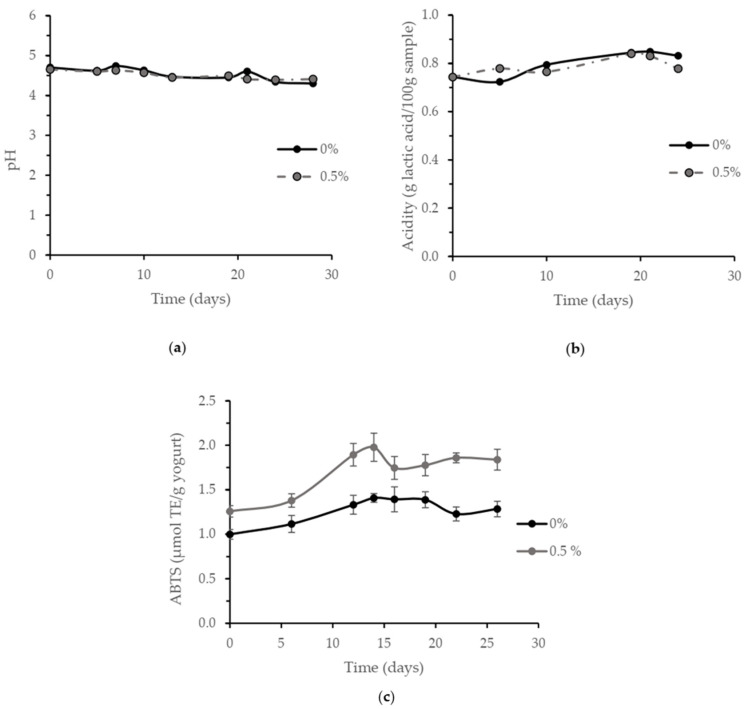
Time course of pH (**a**), titratable acidity (g lactic acid/100 g yogurt) (**b**), and ABTS (µmol TE/g yogurt) (**c**) during storage in the fridge for 28 days of yogurts without (control) and with addition (0.5%) of TGS. Error bars represent standard deviation.

Shelf-life studies were performed by determining pH (Figure 2a) and titratable acidity (Figure 2b) over time (28 and 25 days, respectively). The lifespan of the different yogurt formulations (0 and 0.5% *w*/*w* addition of TGS) was measured by determining pH and titratable acidity, finding no significant variation during the study period (Tukey test, *p* > 0.05) as well as no significant differences between formulations for pH measures at each measure time (Tukey test, *p* > 0.05). Titratable acidity was affected by the incorporation of 1% *w*/*w* of Tannat grape skin, showing lower acid values (data not shown). In agreement with our results, a yogurt formulation fortified with Pinot Noir grape pomace showed a decrease in pH, and titratable acidity was increased during storage [4], which may be related to microbial growth. In the same way, a yogurt added with a red grape skin extract showed pH decrease and titratable acidity increase during 21 days of storage [46].

However, the different yogurt formulations obtained in the current work presented microbial counts (fungi and coliforms) below the limit established in the National Bromatological Regulation (Uruguay) [47] for yogurts, being suitable for its consumption for 28 days (data not shown). These results indicated the preservation of food quality during TGS yogurt shelf-life, showing similar behavior to control yogurt.

The antioxidant capacity of the selected food was followed over time by ABTS method during shelf-life (Figure 2c) (from the day after fermentation), showing increased antioxidant capacity with respect to the control yogurt (formulation without TGS addition). In addition, the antioxidant capacity increased slightly with time until day 12 and then remained unchanged up to the end of the study (28 days). Increased antioxidant capacity during storage may be due to bacterial metabolic activity which could have caused a breakdown of macromolecules with electron transfer antioxidant mechanism, in agreement with the results obtained by Bertolino et al. [5]. Particularly, the antioxidant capacity increase may be explained by the release of bioactive peptides by lactic fermentation proteolysis during storage [48]. It would be interesting to further investigate the bioactive peptides responsible for the antioxidant capacity by isolating them from the yogurt matrix.

### 3.5. Consumers’ Sensory Analysis of Healthy Sustainable Snacks

**Figure 3:**
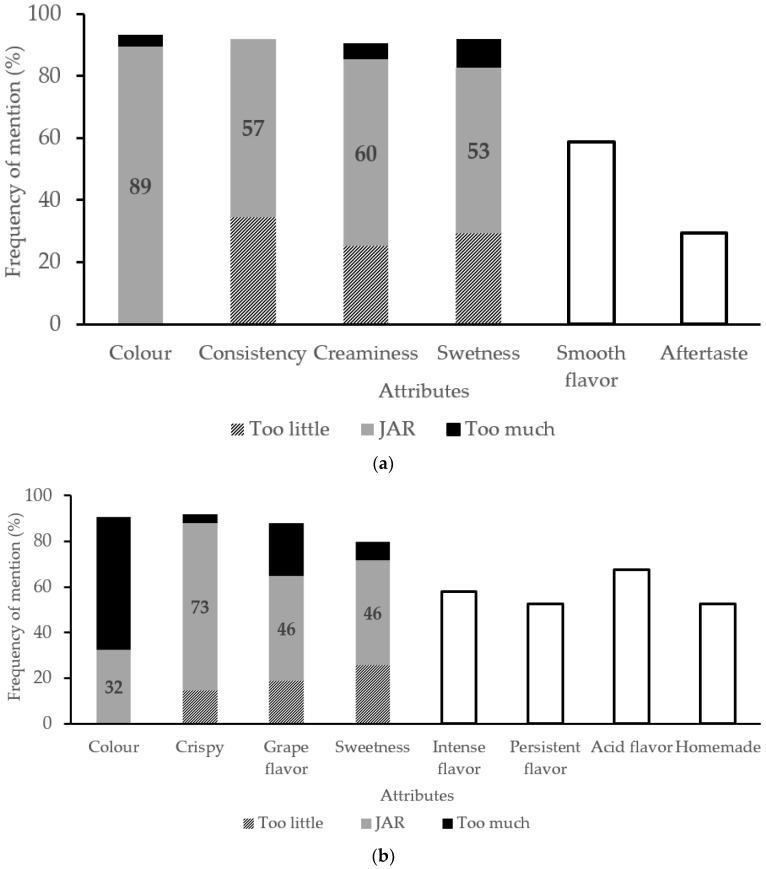
Sensory evaluation study with consumers (*n* = 75) of TGS yogurt (**a**) and TGS biscuits (**b**) using CATA + JAR. Frequency of mention in percentage for just-about-right scores for each dimension (too little, JAR, too much) and each attribute. The number represents the percentage of consumers who selected an attribute as “just about right”. White bars represent the percentage of consumers who selected the shown attributes contributing to the sensory profile of TGS yogurt and biscuit.

Sensory analyses were carried out with informed consumers (*n* = 75) about TGS addition to the developed foods (yogurt and biscuit formulations), obtaining the frequency of mention for the different attributes analyzed through CATA + JAR (Figure 3) and its acceptability evaluated on a scale of 1 to 9.

The evaluated samples consisted of yogurt and biscuits with 0.5 and 20% *w*/*w* of TGS addition, respectively. The acceptability evaluated on a scale of 1 to 9, was 6.3 for TGS yogurt and 5.1 for TGS biscuits. TGS biscuit formulation should be further studied in order to improve acceptability by complementing with inulin, which could mask the off-flavor. As for the frequency of mention of the different attributes analyzed, TGS yogurt (Figure 3a) was characterized by possessing a suitable color and adequate consistency, soft texture, natural flavor, smooth, rich flavor, adequate creaminess, being from little to adequate sweet. TGS biscuits (Figure 3b) were described as being dry, of adequate crunch, from adequate color to too dark, homemade, with intense and persistent flavor, with adequate grape flavor, sweetness, and acid flavor.

Previous sensory analysis results of yogurt fortified with *Vitis vinifera* L. cv. Pinot Noir red wine grape pomace showed no significant differences in appearance liking and overall liking between control, 1 and 2% *w*/*w* wine grape pomace yogurt samples, but 2% *w*/*w* wine grape pomace yogurt received a lower score on flavor and texture liking [4]. However, the sensory analysis by panelists of a yogurt formulation enriched with a grape pomace aqueous extract from Pinot noir vinified in white, resulted in an overall liking score of 6.2 out of 9.0 with 51% of panelists buying the product and finding extract incorporation in yogurt as a potential antioxidant dietary fiber ingredient [49]. In this last study, purchase intention was similar for both informed and non-informed groups, while the health interest questionnaire resulted in an 84% of the volunteers showing medium to high interest for eating healthy, also considering the antioxidant dietary fiber addition to yogurt a good or great idea [49]. Interestingly, these results were similar to those found in the current work. The incorporation of TGS bioactive compounds as an extract could be a good strategy to improve the sensory acceptability while increasing their incorporation.

Regarding biscuits, anthocyanins sources such as TGS have also been studied by raspberry pomace addition to cookies in order to increase fiber content, finding no negative influence on organoleptic characteristics of the product being accepted by consumers with increasing sour taste and less perceptible sweet taste with increasing addition [50]. The biscuit results of the present work agree with the latter work. White grape skin pomace has also been incorporated to cookies formulation finding no negative effect on sensory quality of cookies with 15% of composite flour replacement [51], which is lower than the replacement of the present work.

Both food products developed showed the typical strong color of Tannat grapes, which could also be applied as a natural food dye [3]. In view of consumers’ concern about the toxicity, allergic reactions, and side effects of synthetic food dyes, as well as the concern for health-promoting foods consumption, Tannat grape skin could represent an important source of natural pigments with health benefits [3].

It is important to highlight the need for considering all the factors and multidisciplinary work on the development of food products with improved nutritional value by the addition of natural sources of bioactive compounds and dietary fiber such as TGS, in order to achieve good sensory quality and health claims. Value co-creation is also important to highlight as a means of collective work (study and learning process) between academia, food industry, and consumers in order to obtain foods that satisfy the demands of the consumers from the holistic point of view.

Considering the results as a whole, TGS yogurt presented a higher acceptability than TGS biscuit formulation as well as greater frequency of mention for positive attributes and selected as “just about right”. Consequently, the bioaccessible fraction of TGS yogurt formulation (0.5% *w*/*w*) was selected for further cell studies. The biscuit formulation must be optimized for better sensory results.

### 3.6. Intracellular Effects of TGS Yogurt

Intestinal (IEC-6 and CCD-18Co) and macrophage (RAW264.7) cells MTT assays showed >80% of cell viability for all the tested concentrations (100–10,000 µg/mL of digested TGS yogurt) (data not shown).

**Figure 4:**
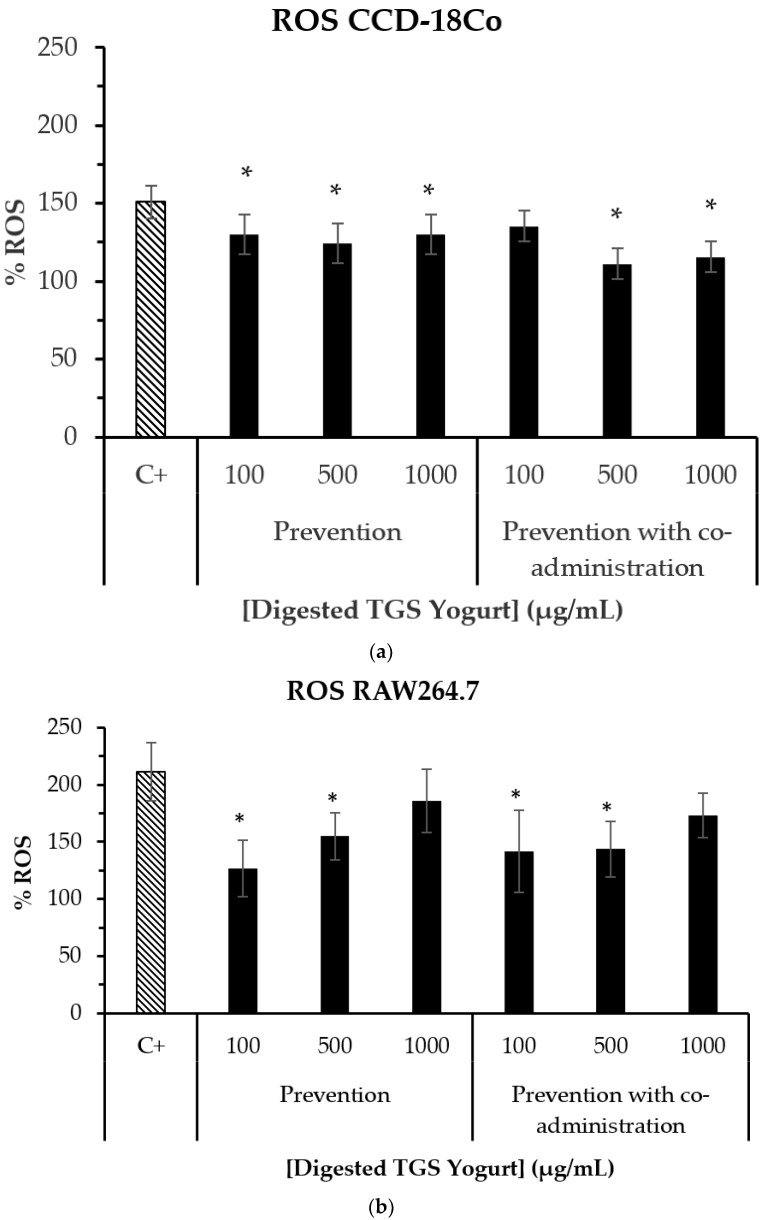
Induced ROS formation in healthy human colon cells (CCD-18Co) (**a**) and mouse macrophages (RAW264.7) (**b**) treated with 0.5% TGS yogurt digest prior to the oxidative damage. Positive control (C+): cells treated with tert-butyl hydroperoxide (1 mM). Prevention assay: cells were treated with samples for 24 h followed by administration of tert-butyl hydroperoxide for 30 min. Prevention with co-administration assay: cells were treated with samples for 24 h followed by co-administration of tert-butyl hydroperoxide and sample for 30 min. Bars represent mean values while error bars indicate standard error of the mean (SEM). * Indicates statistical differences between mean values of the samples (Tukey test, *p* < 0.05) when compared to the positive control (C+).

Induced ROS formation by t-BOOH in healthy human colon cells (CCD-18Co) (Figure 4) was significantly inhibited (*p* < 0.05) by digested TGS yogurt when compared to the control (cells treated with t-BOOH). This result may be associated with the Tannat grape skin anthocyanins constituting the yogurt that have been previously reported for exerting health promoting properties on cells confirming their absorption [27,52]. However, in healthy mouse small intestinal cells (IEC-6), no inhibition was observed (data not shown). Digested TGS yogurt inhibited t-BOOH-induced intracellular ROS formation in RAW264.7 macrophages at the lower concentrations (100 and 500 µg/mL yogurt digest). These results are in agreement with the bioaccessibility of TGS yogurt antioxidants determined in the present work and also with our previous findings on the antioxidant effect of Tannat grape skin bioaccessible compounds on cells [11]. Among polyphenols, anthocyanins have been found to be bioavailable [53] and inhibit intracellular ROS formation under oxidative stress-induced conditions in intestinal cells [54,55]. Other compounds, such as dairy bioactive peptides, that may be released during fermentation and digestion of the yogurt [56], might also play a role on the displayed intracellular antioxidant effect which have been reported for their intestinal absorption and impact on gut health [57].

To the best of our knowledge, this is the first study on intracellular ROS formation in the presence of a digested byproduct-enriched yogurt studied on cell culture.

**Figure 5:**
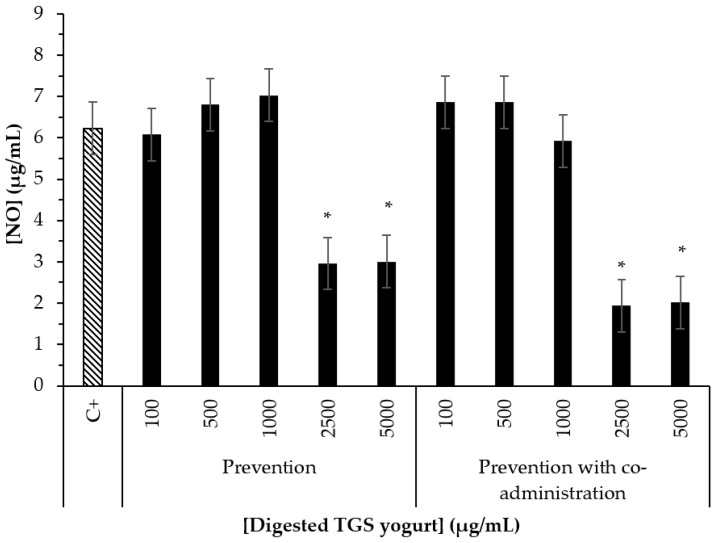
Effect of 0.5% TGS yogurt bioaccessible compounds on NO production in RAW264.7 macrophages. Positive control (C+): cells treated with LPS (1 µg/mL). Prevention assay: cells treated with samples during 24 h followed by administration of LPS during 24 h. Prevention with co-administration assay: cells treated with samples during 24 h followed by co-administration of LPS and sample during 24 h. Bars represent mean values while error bars indicate standard error of the mean (SEM). * Indicate statistical differences between mean values (Tukey test, *p* < 0.05) when compared to the control (C+).

Moreover, a significant marked reduction of NO formation in LPS-induced macrophages was observed for TGS yogurt digest at 2500 and 5000 µg/mL (Figure 5), indicating anti-inflammatory potential. This potential may be associated with the Tannat grape skin anthocyanins (shown by UHPLC-MS/MS results) constituting the yogurt [58,59]. The results of the current work agree with our previous studies on the anti-inflammatory potential of the bioaccessible fraction of a Tannat grape skin extract [11]. As well, dairy bioactive peptides from fermented milk have also been reported for exerting an anti-inflammatory response [60]. To the best of our knowledge, this is the first time that the anti-inflammatory activity of a dairy product added with a grape byproduct after in vitro simulation of digestion is determined through cell studies.

Food prototypes with nutrition claims and potential health promoting properties for having a positive influence in key events involved in diabetes have been obtained. Our preliminary results are very relevant for the reformulation of the snacks with the final aim to achieve products satisficing consumers’ demands for their commercialization. To overcome this limitation of our study, co-creation experiments will be carried out in the future for the reformulation of the foods taking into account the information on the bioaccessibility of bioactive compounds and the sensory profile hereby described. The new formulation of the yogurt should include higher concentrations of TGS and a different presentation with the final aim to achieve a product that is ready to eat, providing health promoting compounds in physiological concentrations and high sensory quality. In the case of the biscuits, the sensory profile should be improved, adding new ingredients to simultaneously mask the off-flavor and enhance the nutritional value [61]. The incorporation of the TGS as a food ingredient is one of the main strengths of the present investigation [62]. The dietary fiber composing TGS seems to naturally protect the bioactive compounds from degradation. The use of TGS avoids the generation of further wastes and reduces the cost of the ingredient [62].

The data on the effects of bioaccessible compounds from TGS sustainable snacks on oxidative stress and the modulation of carbohydrases was confirmed; highlighting their application as health promoting foods is another fundamental strength of the present study. TGS yogurt showed health promoting intracellular effects after digestion, giving added value to the developed food, along with the advantage of using a byproduct of the agro-food industry, with a subsequent decreased environmental impact. Although cell culture studies represent a valid estimate as to what happens at the body level, in vivo studies should still be carried out to confirm the effects in the organism, such as already existing functional yogurt [63], and to discover the underlying mechanisms of action for which TGS yogurt exerts the shown health promoting effects. Furthermore, studies regarding the individual contribution of the bioactive compounds on the exerted bioactivities after in vitro simulation of digestion should be addressed.

In sum, the current study is of great interest because it clearly outlines the strategy to follow in order to obtain high-quality products based on TGS, contributing to nutritional security.

## 4. Conclusions

TGS polyphenolic profile assessed by ultra-high performance liquid chromatography tandem mass spectrometry (UHPLC-MS/MS) showed quercetin-3-galacturonide and quercetin as the main flavonoids, gallic acid as the main phenolic acid, and malvidin-3-pyranoside as the main anthocyanin. TGS digest showed decreased TPC, ABTS, and ORAC-FL antioxidant capacity and increased α-glucosidase inhibition capacity (*p* < 0.05). Two healthy sustainable snacks were developed with the nutritional claims “no-added sugars” and “source of fiber”. Regarding the bioaccessible compounds of the healthy sustainable snacks, digestion enhanced their bioactive properties possibly because of the release of bioactive peptides from milk and wheat proteins. Biscuits showed a significant increase (*p* < 0.05) in ABTS antioxidant capacity while yogurt showed increased α-glucosidase inhibition capacity by the addition of TGS (*p* < 0.05).

Yogurt shelf-life parameters (pH, titratable acidity, and ABTS) were maintained for 28 days. The use of TGS as a food ingredient in biscuits and yogurt was supported by consumers’ acceptance with scores of 5.1 and 6.3, respectively (scale 1–9) and by the sensory profile categorization as “just about right” for most of the evaluated attributes.

TGS yogurt digest showed inhibition of intracellular ROS formation under tert-butyl hydroperoxide-induced conditions in CCD-18Co colon cells (500 and 1000 µg/mL) and RAW264.7 macrophages (100 and 500 µg/mL). Moreover, TGS yogurt digest showed anti-inflammatory potential by a marked reduction of nitric oxide production in pre-treated RAW264.7 macrophages under LPS-induced conditions (2500–5000 µg/mL).

In conclusion, the in vitro bioactivity exhibited by TGS yogurt and biscuits bioaccessible compounds confirm its feasibility for the development of healthy sustainable snacks and its ability to modulate key biochemical events of diabetes pathogenesis. Further studies regarding yogurt formulation to add higher amounts of TGS, as well as biscuit formulation to obtain higher acceptability should be assessed. The role of bioactive peptides released during digestion should also be further studied.

## Figures and Tables

**Table 1:** Yogurt and biscuit formulations.

Yogurt Formulations (g)			Biscuits Formulations (g/100 g Dough Mix)		
Ingredients	Control	TGS	Ingredients	Control	TGS
UHT whole fluid milk (mL)	800	800	Butter	10	10
Skim milk powder	16	16	Sunflower oil	4.25	4.25
Modified cassava starch	4	4	Egg	14	14
Gelatin	4	4	Baking powder	0.5	0.5
CRL inulin (soluble fiber)	10	10	Salt	0.08	0.08
Stevia	0.32	0.32	Sweetener	4	4
Byproduct	0	4 (0.5%)	Wheat flour	67.17	47.17
YO-MIX 495 LYO ferment	250 DCU		Byproduct	0	20

**Table 2:** Data on identification of phenolic compounds composing TGS analyzed by UHPLC-MS/MS.

Negative ESI				
Compound ^1^	TGS ^2^	RT [min]	[M-H]^−^ (*m*/*z*)	Fragments (*m*/*z*)
3-Phenyllactic acid	0.0268	10.6	165.0559	147.0455, 119.0504
Salipurposid	0.0019	11.8	433.1154	271.0607, 151.0041
Astragalin isomer 1	0.0027	11.1	447.0944	284.0334, 227.0356
Astragalin isomer 2	0.0071	11.2	447.0949	284.0334, 227.0356
Caffeic acid	0.0113	9.5	179.0354	135.0455
cis-Aconitic acid	0.0673	2.8	173.0094	129.0197, 85.0297
Eriodictyol	0.0021	13.6	287.0570	151.0041, 135.0456
Gallic acid	0.0990	3.7	169.0145	125.0247
Isorhamnetin	0.0311	14.9	315.0519	300.0283, 151.0037
Myricetin	0.0473	12.3	317.0310	178.9989, 151.0040
Quercetin-3-galacturonide	0.1190	10.7	477.0685	301.0361, 151.0039
Quercetin	0.1181	13.5	301.0361	151.0040, 107.0141
Quercetin-3β-D-glucoside	0.0578	10.7	463.0900	300.0282, 271.0254
Syringic acid	0.0003	11.2	197.0459	182.0225, 123.0091
Vanillic acid	0.0007	10.8	167.0352	152.0118, 123.0091
Vanillyl alcohol	0.0073	5.2	153.0561	138.0325, 123.0091
Naringenin	0.0036	14.8	271.0620	151.0041, 119.0505
**Positive ESI**				
**Compound ^3^**	**TGS ^2^**	**RT [min]**	**[M]^+^ (*m*/*z*)**	**Fragments (*m*/*z*)**
Cyanidin 3-(6-O-acetylglucoside)	0.00011	9.8	491.1184	287.0550
Cyanidin-3-O-(6-p-coumaroyl) glucoside	0.00032	10.9	595.1446	287.0550
Cyanidin-3-pyranoside	0.00021	8.5	449.1078	287.0550
Delphinidin-3-(6-O-acetylglucoside)	0.00014	9.2	507.1133	303.0500
Delphinidin-3-O-(6-p-coumaroyl) glucoside	0.00087	10.5	611.1395	303.0500
Delphinidin-3-pyranoside	0.00094	7.8	465.1027	303.0500
Malvidin-3-(6-O-acetylglucoside)	0.01076	10.4	535.1446	331.0800
Malvidin-3-O-(6-p-coumaroyl) glucoside	0.01766	11.5	639.1708	331.0800
Malvidin-3-pyranoside	0.02569	9.2	493.1340	331.0800
Peonidin-3-(6-O-acetylglucoside)	0.00105	10.4	505.1341	301.0700
Peonidin-3-O-(6-p-coumaroyl) glucoside	0.00194	11.5	609.1603	301.0700
Peonidin-3-pyranoside	0.00206	9.2	463.1235	301.0700
Petunidin-3-(6-O-acetylglucoside)	0.00134	9.9	521.1290	317.0700
Petunidin-3-O-(6-p-coumaroyl) glucoside	0.00294	11.0	625.1552	317.0700
Petunidin-3-pyranoside	0.00013	11.2	479.1184	317.0700

^1^ Compound Discoverer 3.1 (mzCloud library, Advanced Mass Spectral Database). ^2^ Results normalized with TIC area (area/area TIC). ^3^ Identified as proposed by Ivanova et al. [26].

**Table 3:** Results of antioxidant and antidiabetic bioaccessible compounds estimated by analysis of total polyphenol content (TPC), antioxidant capacity by ABTS and ORAC-FL, α-glucosidase and α-amylase inhibition capacities.

Analysis	TGS	TGS Digest
TPC (mg GAE/g sample)	29.85 ± 2.20 ^b^	7.41 ± 0.50 ^a^
ABTS (µmol TE/g sample)	28.28 ± 1.27 ^b^	18.13 ± 2.05 ^a^
ORAC-FL (µmol TE/g sample)	150.3 ± 11.1 ^b^	128.3 ± 13.3 ^a^
α-glucosidase (IC_50_, mg/mL)	11.67 ± 0.71 ^c^	8.23 ± 0.44 ^b^
α-amylase (IC_50_, mg/mL)	11.65 ± 0.11 ^b^	102.80 ± 8.93 ^c^

TGS: Tannat grape skin powder. IC_50_: half maximal inhibitory concentration. Results are expressed as mean values ± SD (*n* = 3). Different letters indicate significant differences (Tukey, *p* < 0.05) between values in the same row. Sample solutions were prepared in triplicate and assayed in triplicate.

## Data Availability

We do not have supplementary data to show other than the results presented in the “Results and Discussion” section.
